# Authoring the identity of learner before doctor in the figured world of medical school

**DOI:** 10.1007/s40037-017-0399-0

**Published:** 2018-01-05

**Authors:** Evangeline Stubbing, Esther Helmich, Jennifer Cleland

**Affiliations:** 1 School of Medicine, Medical Sciences and Nutrition, University of Aberdeen, Foresterhill, Aberdeen, UK; 2 Center for Education Development and Research in Health Professions, University Medical Center Groningen, Groningen, The Netherlands; 3 Centre for Healthcare Education Research and Innovation, Institute of Education for Medical and Dental Sciences, University of Aberdeen, Foresterhill, Aberdeen, UK

**Keywords:** Medical students, Qualitative research, Professional identity formation, Emotion, Figured worlds

## Abstract

**Introduction:**

Students enter the ‘figured world’ of medical school with preconceptions of what it means to be a doctor. The meeting of these early preconceptions and their newly developing identities can create emotional tensions. The aim of this study was to advance our understanding of how such tensions were experienced and managed. Using figured worlds as a theoretical framework we explored students’ interactions of preconceptions with their newly developing professional identities in their first year at medical school. Advancing our understanding of this phenomena provided new insights into the complex process of identity formation.

**Methods:**

This was a qualitative study underpinned by a constructivist epistemology. We ran biannual focus groups with 23 first year students in one UK medical school. Data were recorded, transcribed and then template analysis used to undertake an inductive, iterative process of analysis until it was considered the template provided a detailed representation of the data.

**Results:**

Significant preconceptions associated with the identity of a doctor were ‘to help’ and ‘to be a leader’. These early preconceptions were in conflict with realities of the figured world of medical school creating the emotional tensions of ‘being unable to help’ and ‘lacking power’, with implications for interactions with patients. By the end of year one students’ negotiated tensions and ‘self-authored’ their identity as a learner as opposed to an imagined ‘as if’ identity of a doctor.

**Discussion:**

We revealed how preconceptions associated with becoming a doctor can conflict with a newly developing professional identity highlighting the importance of supporting students to embrace the formation of a ‘learner’ identity, a necessary part of the process of becoming a doctor.

## What this paper adds

Students enter the ‘figured world’ of medical school with preconceptions of what it means to be a doctor. The meeting of these early preconceptions and their newly developing identities can create emotional tensions hindering rather than helping the development of a professional identity. We revealed how these preconceptions conflict with a newly developing professional identity throughout the first year of medical school. Our findings highlight the importance of educators supporting students to embrace the formation of a ‘learner’ identity early on in medical school, as this is a necessary part of the process of becoming a doctor.

## Introduction

Identity formation is about how people come to make sense of themselves, how they ‘figure’ out who they are as they interact with the ‘world’(s) they are part of and with others who both exist within and outside of these worlds [1, 2]. Medical students are positioned between worlds, between being adolescents [3] in the lay world of patients and becoming doctors and professionals [4]. A consequence of this transitional position is that medical students are continually *negotiating *or* authoring *their identities in terms of who they think they are and how they portray themselves to others in relation to their training and as future doctors [5].

Research to date has illuminated the *external *social factors within the world of medical education implicated in identity formation: for example, socialization into the culture of medical school [6–8] or the emotional experience of patient encounters [9–11]. More recent studies have explored the influence of *internal* factors. These internal factors include the early preconceptions or imaginations that medical students associate altruistically with the identities of a doctor (such as to help people and cure the sick) [4, 12–15], but also less desirable preconceptions (for example to possess power and status) [13–15]. As students try to ‘figure’ who they are within the world of medicine their early preconceptions may be reinforced (beneficial ‘consonance’), or contested (emotionally conflicting ‘dissonance’) [16, 17]. As a consequence of the experience of dissonance students may doubt their world-views, aspirations, question their self-worth and may then struggle to succeed in meeting the requirements of their profession [16]. It is clear, therefore, that this continual negotiation of a professional identity has an emotional cost, yet the emotions involved in identity formation are often overlooked in medical education research [18]. Moreover, where there is tension or dissonance as students attempt to integrate their early preconceptions about being a doctor with their developing identities once at medical school (for example; when being unable to cure the sick [4] and when facing the expectation that a clinician should be emotionally detached) [19, 20], early preconceptions may hinder rather than help students form their professional identities.

If medical students are not entering medical school as ‘blank slates’, it is crucial to explore further how such internal tensions and associated emotions are experienced and managed. Advancing our understanding of this phenomena will provide new insights into the complex process of identity formation and assist educators in how best to support students through this experience.

Thus, and responding to calls to acknowledge the role of emotion within medical education [17, 18, 21], our aim was to explore the emotions medical students experience when starting to form new identities early in medical school. We used ‘figured worlds’ theory [1] to help make explicit the links between identity formation and emotions [22], and to help us explain our findings in a coherent way.

## Methods

### Study paradigm/methodology

This was a qualitative, interpretative study underpinned by a constructivist epistemology [23].

### Context

The study took place at one medium-sized UK medical school offering a five-year undergraduate program (the norm in the UK). The program is systems- and case-based, with early clinical exposure to acute hospital wards and a patient home visit in the first year.

### Participants

In 2013, all 169 first year medical students were invited to participate in a five-year longitudinal study via a whole-class presentation, poster advert, and class email [24]. Twenty-three students self-selected to take part in the study [25] (14 female and 9 male participants aged between 17 and 26 years). This paper reports on the first year of this study.

### Data collection

We used focus groups for data collection to allow participants to lead responses, discussion [26], and also to stimulate potentially inaccessible thoughts [27]. The 23 participants were formed into four focus groups on the basis of optimal group size [23]. Two groups of six, one group of five and a group of four were formed. Two participants unable to attend with the group of four were interviewed later as a pair. All participants attended and remained in the same groups/pairs throughout the study. Data collection was longitudinal with two sets of focus groups: the first within a few weeks of starting medical school and the second at the end of Year 1. Focus groups, lasting around 1 h, were undertaken on medical school premises. Participants received light refreshments but no other incentives. ES conducted the focus groups using a semi-structured and open-ended question guide to elicit participants’ experiences and emotions [28]. Developed from existing literature the questions in the first set of focus groups asked about reasons for studying medicine and expectations of forthcoming experiences. In the second set of focus groups, these reasons were re-visited, experiences explored, and future thoughts elicited.

### Data management and analysis

Focus groups were audio recorded and transcribed verbatim, with names removed, to engage anonymously with textual data [29]. We conducted template analysis (TA) [30, 31] using analysis software NVivo 10 (http://www.qsrinternational.com/products_nvivo.aspx) for data management and coding. The lead researcher (ES) undertook the process of preliminary coding. These codes were then discussed by the author team and used to develop two separate ‘coding templates’, one for each set of focus groups, the first round (October 2013) and second round of focus group data (June 2014) to conduct analysis both across the set of focus groups and longitudinally. These templates formed the foundation and means of organization for recurrent features of the participant accounts [32]. Coding and analysis were iterative and inductive: with team discussions at regular intervals to modify the templates until we had a rich representation of the data [33].

Interpretation of qualitative data is dependent upon the researcher standpoint and the social context in which the research is undertaken [33]. From a constructivist perspective, research outcomes will never separate from the biases, assumptions and the characteristics of the researcher [34]. Thus, throughout the study, ES maintained a reflexive diary and as a team we regularly considered our stances and possible biases recognizing how these may be influential to the research process and findings. ES was known to participants as a Clinical Skills Facilitator during the time of the study. EH is a medical doctor holding a PhD in medical education who works as a clinical supervisor. Having the experience of becoming a doctor herself made it easy for her to relate to the stories of students, but at the same time, this specific background may have brought a certain bias to her perception of the experiences of students. JC is a clinical and occupational psychologist working in medical education research. Her background and education means she has no personal knowledge of professional identity formation in medicine, and her interest in this topic is quite neutral.

## Theoretical framework

Holland et al. broadly defined figured worlds as ‘socially produced, culturally constituted activities’ (P. 40–41) where people produce (perform) new self-understandings (identities). Figured worlds are sites of individual possibility, or ‘as if’ world(s) but Holland et al. also make clear that because figured worlds are culturally and socially based, they are mediated by relations of power, status, and rank, implicated through daily activities. In other words, within these figured worlds, or contexts, certain social acts have significance and people’s positions matter.

Each figured world is organized by socially constructed master narratives or preconceptions on which the world is based (such as doctors should be able to cure the sick). Day-to-day social practices and activities are interpreted against these narratives. Figured worlds are constructed and reconstructed through daily actions and a process of ‘positionality’; that is, the positions ‘offered’ to people in a certain figured world (such as patient, doctor or medical student) which they may accept, reject, or negotiate. This is referred to in figured worlds theory as the ‘space of authoring’, or of people answering and responding by accepting, rejecting or negotiating the identities offered to them. In other words, people ‘self-author’ who they are through activities that they attach significance to, in relation to the social types that populate these figured worlds and in social relationships with the people who perform these worlds.

Figured worlds can create a challenge or tension when they are different to what was originally constructed, as in preconceptions, for example, that being a doctor is not only about curing the sick but is also about providing support to ensure a comfortable and dignified death. In response to such tensions, ‘self-authoring’ may take place, where individuals use their social resources to craft a response, to re-write themselves into the world [1].

Following the recognition of its value in educational research [2, 35–37] and emergent use in the medical education field [38–41], Holland et al.’s ‘figured worlds’ theory has been promoted to explore professional identity formation in medical education [41].

## Ethics

Ethical approval was granted by the local College Ethics and Research Board (CERB). Participants were provided with verbal and written assurance of anonymity, confidentiality and data security.

## Results

Template analysis revealed three overarching themes and associated subthemes: preconceptions of being a doctor, e. g. to help/make a difference and to be a leader. Tensions experienced with preconceptions included being unable to help/make a difference, feeling a sense of pressure to help/make a difference and lacking power. Moreover, analysis revealed participants negotiating tensions and self-authoring identities.

Data excerpts are presented in the following format i. e. ‘Female Participant 1’ (FP1)/‘Male Participant 8’ (MP8) representing anonymized participant identifiers, ‘Focus Group 1’ (FG1), representing which focus group the excerpt originates from and Time 1 (T1), start of Year 1 or Time 2 (T2), end of Year 1 at medical school representing when the focus group was undertaken.

## Preconceptions of being a doctor

On arrival to medical school, as part of their early identity formation participants had formed a number of preconceptions associated with the figured world of medicine and the position of a doctor. Two significant preconceptions were expressed. The first somewhat altruistic, participants considered ‘to help/make a difference’ was fundamental to being a doctor: *‘I think my angle will be trying to be something like a doctor without borders or something like that, and so the idea of helping the helpless’* (MP11-FG2-T1). Second, participants considered that a doctor is ‘to be a leader’: ‘*But I think to make a real difference, you have to be a doctor because they’re the team leader; they make the decisions and everything follows down from them’ *(FP2-FG1-T1).

## Tensions experienced with preconceptions

In contrast to their preconceived figured world of a being a doctor to ‘help/make a difference’ and a ‘to be a leader’, the realities of the newly experienced world of medicine created an evident tension (dissonance).

Participants throughout their first year discussed ‘being unable to help/make a difference’ with reference to a lack of knowledge: *‘I’ll still feel like I’m an inexperienced first year medical student and I can’t do anything and I don’t have the knowledge to do anything’* (FP16-FG3-T1). And *‘We were really annoyed by the, uh, fact that I don’t know much about it, or don’t know how to treat it (referring to a patient condition) …’* (MP10-FG2-T2).

At the end of Year 1 participants considered that, despite lacking the knowledge and thus being unable to help/make a difference, patients still granted them their trust. This created ‘feeling a sense of pressure to help/make a difference’ and added to the tension. *‘It’s that trust that you know what you’re doing, when in actual fact, you probably don’t [laughs]’* (MP8-FG2-T2). Or: *‘… putting so much trust in a single person can add pressure to that person too and if you mess up then you’ll feel a million times worse because that patient really trusted you’ (*FP17-FG3-T2).

Throughout their first year participants expressed a tension between their position as a first year medical student and their earlier preconception of being a leader in the figured world of medicine. This tension was expressed through their recognition of ‘lacking power’: ‘*You realize you don’t have as much power as you think’ *(MP8-FG2-T1). A perceived lack of respect also contributed to the tension of ‘lacking power’: *‘I got told … you’re a little girl no one will ever respect you’* (FP5-FG1-T2).

## Negotiating tensions and self-authoring

As the participants journeyed through Year 1 at medical school, they negotiated the tensions outlined above through a process of self-authoring. By the end of Year 1, participants expressed an attempt to negotiate the tension of being unable to help/make a difference by being eager to address their lack of knowledge, a way in which to write one’s self into the figured world of medicine: *‘I kind of feel like I want to be able to know more and start to be able to do more’* (FP4-FG1-T2). At the same time, participants realized that addressing this tension required reconstructing their position, by recognizing and accepting they cannot know everything at this stage: *‘It’s only human. We, we can’t know everything’* (MP7-FG2-T2).

By the end of Year 1 participants also recognized that their position as a medical student does grant responsibilities, if not the authority and leadership of a doctor: *‘Maybe, now that you’re, like, a medical student … you can have, not authority, but you’ve got responsibility …’* (MP9-FG2-T2). Participants look to the year ahead imagining a stronger position that might enhance the notion of being a leader:* ‘I think second year has more of a standing, people are like, oh they’ve got some medical knowledge, they know what they’re doing’* (FP1-FG1-T2).

## Discussion

We found that, on entering the figured world of medicine, students experience tensions between their positional identities as medical students and their earlier internal preconceptions or imaginations of being doctors who help/make a difference and are leaders. Instead, being ‘just’ first year medical students they found themselves lacking both the knowledge and power to be able to help/make a difference and to lead. By the end of the first year of medical school, although still lacking knowledge and power, our students appear to resolve the tensions they had experienced, learning to acknowledge limitations of knowledge and power, yet recognized that being a medical student granted them privileges and responsibilities (but not yet authority). Instead of focusing only on the ‘as if’ or imagined identities of being a doctor, they re-aligned their position into the self-authored identity as learner and first year medical student. The very identity as a first year medical student, nothing more, nothing less, granted our students a legitimate position, which in turn allowed them to enter the world of medicine in which they could further develop their future professional identities.

Being trusted by patients felt inappropriate to our participants. Trust is fundamental to the status of a professional, and granted as part of a social and moral contract between patients and doctors [42, 43]. Yet, year 1 students felt unable to fulfil their end of this ‘contract’, because of lacking necessary knowledge and skills. This resulted in a tension, articulated as a sense of pressure. This reflects the findings of earlier studies: for example Lingard et al. (2003) identified tensions in third year medical students, concerning uncertainty and limitations of knowledge during case presentations ([44], see also [45]).

In the eyes of our participants, trust needs to be gained, by means of competence (having enough knowledge), in their wish to take care for people (helping patients, making a difference), or through the position or power granted to doctors enabling them to make a difference. Two of these ‘discourses’ were previously described by MacLeod (2011), when she explored how students may experience tensions between the dominant discourse of competence, and the less valued discourse of caring [20]. Our study adds a third issue that might be in play when students negotiate ‘as if’ identities, one which might be called a discourse of power. As far as we know, our study is among the first showing that power is already part of the preconceptions students have as early as entering medical school, and that lacking power is central to the tensions they experience in the first year of medical school. Previous studies have identified ‘power’ as a negative preconception [13–15]. However, for our participants power seemed less about assuming a dominant and superior position over others, or having status per se, and more as a means to make a difference. This merits further investigation.

This study has strengths and limitations. The study was conducted in one medical school so the results may not be transferable given the importance of context in identity formation (for example, factors contributing to preconceptions may differ for instance as a result of family obligation attitudes [46] and core values varying by country and culture [40]). This requires further investigation. Our longitudinal approach to data collection enabled deeper understanding and new insights into the complex process of identity formation [47, 48]. In particular, this approach enabled an insight into the early preconceptions to be a leader and how this was experienced within the first year of being a medical student. Following up participants even further into medical school would enable the opportunity to see how their preconceptions change over time, and would also enable further insight into how they experience the expectation of trust as they move nearer to the figured world of a doctor. One of the potential limitations of the study is that, despite attempts to minimize contact, (i. e. not teaching or assessing the participants directly), interviewees may not have felt comfortable to be open and discuss matters with someone they perceived as a ‘teacher’ and part of their medical school hierarchy. However, we reflected on this as a team at regular intervals and our impressions was that this was not an issue in practice.

Studies using figured worlds theory in medical education have typically employed discourse [22, 40] or linguistic forms of analysis [38]. However, the combination of figured worlds theory and other methodologies, as in our study, has been successfully utilized in the wider literature [35–37]. This methodological ‘importing’ (the transference of concepts and tools from one field where it is well known to another field where it is less known) is valuable because it provides opportunities for addressing old problems in new ways [49].

The tension of not having enough knowledge and power to help suggests the importance of helping students to affirm a stronger sense of their positional identity as a learner as opposed to an imagined ‘as if’ identity of a doctor. This may be supported through increasing students’ awareness of the process of identity formation in their first months of medical school, and encouraging reflection on this process [50]. Raising awareness of the process of identity formation through personal reflection may help students to work through dilemmas and tensions associated with early internal identities and preconceptions. This may enhance positive development for a professional identity [19].

## Conclusion

This paper explored participants’ internal preconceptions associated with becoming a doctor. We revealed how these preconceptions can conflict with a newly developing professional identity throughout the first year of medical school. Our findings highlight the importance of supporting students to embrace the formation of a ‘learner’ identity early on in medical school, as this is a necessary part of the process of becoming a doctor.
